# Interactions of Transition Metal Dichalcogenide Nanosheets With Mucin: Quartz Crystal Microbalance With Dissipation, Surface Plasmon Resonance, and Spectroscopic Probing

**DOI:** 10.3389/fchem.2019.00166

**Published:** 2019-03-29

**Authors:** Boshi Liu, Tao Yu, Renliang Huang, Rongxin Su, Wei Qi, Zhimin He

**Affiliations:** ^1^Tianjin Key Laboratory of Indoor Air Environmental Quality Control, School of Environmental Science and Engineering, Tianjin University, Tianjin, China; ^2^State Key Laboratory of Chemical Engineering, Tianjin Key Laboratory of Membrane Science and Desalination Technology, School of Chemical Engineering and Technology, Tianjin University, Tianjin, China; ^3^School of Pharmaceutical Engineering of Traditional Chinese Medicine, Tianjin University of Traditional Chinese Medicine, Tianjin, China; ^4^Collaborative Innovation Center of Chemical Science and Engineering (Tianjin), Tianjin, China

**Keywords:** transition metal dichalcogenide nanosheets, mucin, interactions, quartz crystal microbalance, surface plasmon resonance

## Abstract

Ultrathin 2-dimensional transition metal dichalcogenides (TMDs) have become a class of high-potential materials in biomedicine due to their intriguing properties. They have been applied to solve biomedical challenges, such as biosensing, bioimaging, drug delivery, and cancer therapy. However, studies of the interactions between these materials and biomolecules are insufficient. Mucous tissue serves as a barrier to foreign hazardous substances and a gel layer for substance exchange. The main organic matter of mucous tissue is mucin, so it was selected as a model biomolecule to study its interactions with six different TMD nanosheets (NSs), including single-layered (SL), few-layered (FL), and small few-layered (SFL) MoS_2_ and WS_2_ NSs, using quartz crystal microbalance (QCM) with a dissipation monitor (QCM-D) and surface plasmon resonance (SPR). Additionally, UV absorption, fluorescence, and circular dichroism (CD) spectroscopy were applied to investigate the mechanism of the interactions and to study the conformational change of mucin. We found that the TMD NSs could adsorb on the mucin layer and affect its viscoelasticity. The results indicated that the SL WS_2_ NSs exhibited the highest initial absorption rate and the maximum absorption amount, while the SL MoS_2_ NSs exhibited the highest initial desorption rate. During the adsorption, the viscoelasticity variations of the mucin layer caused by the WS_2_ nanosheets were weaker than those caused by the MoS_2_ nanosheets. Furthermore, the conformational changes of mucin caused by the SL MoS_2_, SL WS_2_, and SFL MoS_2_ NSs were higher than those resulting from other TMD NSs. These findings provide important information on the interactions between TMD NSs and mucin and provide useful insights into the interfacial behavior of TMD NSs before they enter tissues.

## Introduction

Mucus is secreted by specialized goblet cells in the columnar epithelium and serves many functions including lubrication, maintenance of a hydrated layer over the epithelium, a barrier to hazardous substances, and a permeable gel layer for the exchange of gases and nutrients with the underlying cells and tissues (Bansil and Turner, 2006; Linden et al., 2008; McGuckin et al., 2011; Demouveaux et al., 2018). The main organic component that is responsible for the functions is mucin, which is heavily glycosylated with complex oligosaccharides (Hollingsworth and Swanson, 2004; Bansil and Turner, 2006; Feiler et al., 2007; Pelaseyed et al., 2014). Therefore, mucin is closely related to many mucus-related diseases, the intake of nutrients, and the adsorption and delivery of drugs through the mucus barrier (Bansil and Turner, 2006; Boya et al., 2017).

Two-dimensional nanosheets of transition metal dichalcogenides (TMDs) are fundamentally and technologically intriguing (Chhowalla et al., 2013; Tan and Zhang, 2015); in particular, MoS_2_ and WS_2_ have attracted interest as they possess a band gap, which is important for integration into electronic device structures. TMDs have provided great opportunities to solve the challenging problems in biomedicine (Li et al., 2017; Zhang et al., 2018), including but not limited to biosensing (Song et al., 2014; Chen et al., 2017; Singhal et al., 2018), bioimaging (Cheng et al., 2014; Liu et al., 2015; Choi et al., 2017; Song et al., 2017), drug delivery (Liu et al., 2014; Yin et al., 2014; Chen et al., 2017; Dong et al., 2018), and cancer therapy (Cheng et al., 2014; Liu et al., 2015; Qian et al., 2015; Song et al., 2017). However, TMDs could potentially be risky to human health and the environment (Guiney et al., 2015, 2018; Kumar et al., 2017). For example, MoS_2_ has been shown to be toxic toward planktonic cells, biofilms, and mammalian cells in the presence of electron donors (Fan et al., 2015), to bind to the K^+^ channels and disturb their functions (Gu et al., 2018), to activate TGF-beta/Smad pathways and perturb the metabolome of human dermal fibroblasts (Yu et al., 2017), and to exhibit cytotoxicity and impact inflammation (Moore et al., 2017).

Sufficient assessments of the hazards of 2D TMD nanosheets (TMD NSs) should be conducted before the biomedical applications of such novel inorganic nanomaterials. However, there are only a few studies on the interaction between TMD NSs and proteins via molecular simulation (Fan et al., 2017; Xiao et al., 2018). Therefore, in this work, we conducted experimental studies on the interactions between TMD NSs and mucin. As mucin achieves its physiological functions in mucosal tissue as a supported hydrogel layer on the epithelial cells, we coated this glycoprotein onto the gold sensing chip of quartz crystal microbalance (QCM) and surface plasmon resonance (SPR) apparatus to form a hydrogel layer and mimic the mucosa and to investigate its interactions with TMD NSs during the adsorption and desorption. Spectroscopic methods, including UV absorbance spectroscopy, fluorescence quenching spectroscopy, and circular dichroism (CD) spectroscopy were also applied to further verify the results obtained from the QCM and SPR study, investigate the mechanism of the interactions, and study the conformational change of mucin during the interactions.

## Materials and Methods

### Materials

Mucin from bovine submaxillary gland was purchased from Shanghai Yuanye Biotech Co., Ltd, China. TMD NS aqueous dispersions (0.1 mg/mL) were purchased from XFNANO Materials Tech Co., Ltd, and the layered NSs were prepared via the lithium intercalating method. The product information of these TMD NS aqueous dispersions was provided by the supplier and listed in Table 1. The thickness was obtained by AFM and single layer percentage was obtained by TEM. All of the data were acquired via statistics of the results from multiple experiments. All solutions were prepared using ultrapure water (18.2 MΩ·cm^−1^, Sartorius Arium Pro VF, Germany).

**Table 1:** Sample information of TMD NS aqueous dispersions.

**Sample**	**Single-layered MoS_**2**_**	**Single-layered WS_**2**_**	**Few-layered MoS_**2**_**	**Few-layered WS_**2**_**	**Small few-layered MoS_**2**_**	**Small few-layered WS_**2**_**
Abbreviation	SL MoS_2_	SL WS_2_	FL MoS_2_	FL WS_2_	SFL MoS_2_	SFL WS_2_
Thickness (nm)	~1	~1	1–10	1–10	1–10	1–10
Single layer percentage (%)	≥90	≥90	1–10	1–10	1–10	1–10

### Measurement of Size and Electrophoretic Mobility of the TMD NSs

The TMD NS aqueous dispersions were diluted to 0.01 mg/mL with ultrapure water. Thereafter, the hydrodynamic diameter and the electrophoretic mobility (EPM) of the TMD NSs was measured using a zetasizer (Malvern Nano ZS, UK).

### QCM Study

QCM (Q-Sense E1, Biolin, Sweden) was applied to monitor the real-time mass and viscoelasticity change of the mucin layer during its interaction with the TMD NSs. Prior to the experiments, the bare gold chips were cleaned following the protocol in the QCM with a dissipation monitor (QCM-D) manual. Briefly, the gold chips were treated in a UV-ozone cleaner (PSD-UV8, Novascan, USA) for 30 min to destroy the organic matters and then soaked in 75% ethanol and cleaned in an ultrasonic cleaner for 30 min, followed by thorough rinsing with ultrapure water and drying with nitrogen. During the QCM experiments, the flow rate was set as 60 μL/min maintaining a laminar flow in the sensing module. Ultrapure water flowed through the channel at the beginning of each experiment, and after the signal of frequency (*F*) and dissipation (*D*) became stable, samples were added following the stages below. (I) The formation of mucin: 0.2 mg/mL mucin aqueous solution flowed over for ~20 min and coated the bare gold chip, forming a layer of mucin on its surface. (II) The rinsing of the mucin layer: ultrapure water was flowed over the mucin for ~10 min to remove the unstably combined mucin. (III) The interaction of TMD NS and the mucin layer: a 0.1 mg/mL TMD NS dispersion was flowed through the channel for ~45 min, giving a sufficient interaction with the mucin layer. (IV) Desorption: ultrapure water was flowed into the channel again. The signal of F and D at the seventh overtone of each experiment was recorded for the study.

### SPR Study

An SPR apparatus (SPR Navi 200, BioNavis, Finland) was used to real-time monitor the refractive index of the materials during the interactions between the TMD NSs and mucin layer. The bare gold SPR chips were cleaned using the method mentioned in the “2.3. QCM study” session. At the beginning of each experiment, the twelve-way valve was set to “Load,” and the 100 μL sample loops were filled with a 0.2 mg/mL mucin aqueous solution. Ultrapure water flowed as a running buffer at the rate of 5 μL/min to maintain a laminar flow. After the stable signal of the SPR angle was achieved, the 12-way valve was set to “Inject,” and the mucin solution flowed over the bare gold chip and formed a mucin layer on its surface. The twelve-way valve was set to “Load” again after the mucin solution was exhausted, and the sample loops were filled with 0.1 mg/mL TMD NS dispersions. Then, the flow rate was set to 2 μL/min, and the twelve-way valve was set to “Inject,” resulting in a slow flow of the TMD NS dispersions over the mucin and a sufficient interaction with it. The experiment was terminated after the TMD NS dispersions in the sample loops were exhausted.

### The Spectroscopy Studies

UV absorption spectra, fluorescence spectra and CD spectra were applied to investigate the interactions between the TMD NSs and mucin in the aqueous phase. TMD NS dispersions and mucin aqueous solutions were fully mixed and interacted for 2 min at room temperature, and then the spectroscopy spectra of the mixtures were measured. In the UV and fluorescence spectroscopy experiments, the final concentration of mucin was 0.1 mg/mL, while in the CD measurement, it was 0.5 mg/mL. The UV absorption measurements were performed using a TU-1810 spectrophotometer (Persee, China), and the fluorescence spectra were evaluated using a Cary Eclipse fluorescence spectrometer (Varian, USA). A J-810 CD spectrometer (Jasco, Japan) was used for CD spectrum determinations. The TMD dispersions with the same concentration as in the mixtures were used as a reference sample in all of the spectroscopy measurements.

## Results and Discussion

### Size and Electrophoretic Mobility of the TMD NSs

**Figure 1:**
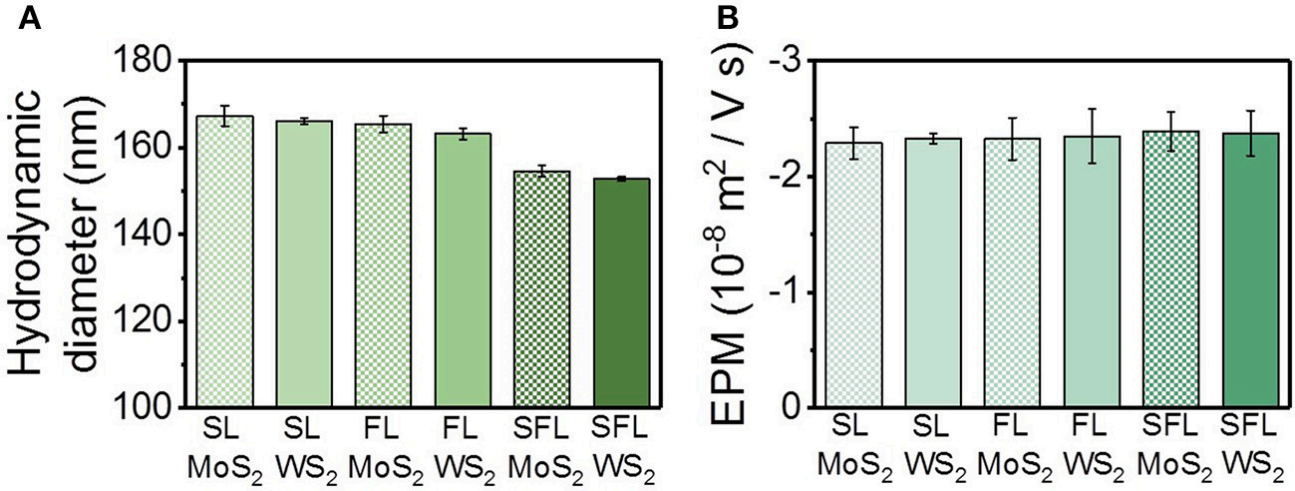
Hydrodynamic diameter **(A)** and electrophoresis mobility **(B)** of the TMD NSs.

The interactions between proteins and nanomaterials are related to the physicochemical properties of the nanomaterials, including size and surface charge (Xia et al., 2015; Sun et al., 2018). Hydrodynamic diameter is the diameter of an imaginary sphere, which has the same diffusion rate as the real particle (Lattuada et al., 2003). The hydrodynamic diameters of the TMD NSs were measured by the Nano ZS Zetasizer based on the principle of dynamic light scattering and the Brownian movement of the sample, and the results were achieved through the calculation by the software through the Stokes-Einstein equation. The hydrodynamic diameters of the TMD NSs were compared in Figure 1A. There was no significant difference in the hydrodynamic diameters of the single-layered and few-layered TMD NSs, although the single layer percentage was different. The hydrodynamic diameters of the small few-layered (SFL) TMD NSs were smaller than others. The TEM pictures of MoS_2_ nanosheets were shown in Supplementary Figure 1 for reference. A sketch (Supplementary Figure 2) was given according to the product information of the TMD NSs, and the results mentioned above to show the morphology of the TMD NSs. Among all the TMD NSs in this study, SL MoS_2_ and SL WS_2_ had the biggest diameters and the smallest thicknesses, while SFL MoS_2_ and SFL WS_2_ were smallest in diameter and biggest in thickness. FL MoS_2_ and FL WS_2_ were in the middle. Herein, “flakiness” is noted to describe “the ratio of diameter and thickness,” so the single-layered TMD NSs were the highest in flakiness, while the SFL TMD NSs were the lowest.

The EPM values of the TMD NSs were compared in Figure 1B. All the TMD NSs exhibited negative charged surfaces and the EPM values of the TMD NSs were very close, indicating the similar mobility under the same electric field.

### The Interactions Between the TMD NSs and Mucin Layer

Mucin usually covers the surface of the epithelial cells of the organisms forming a hydrogel layer (Li et al., 2013; Brandão et al., 2017), which constitutes the main part of the mucosal structure. A mucin layer, which mimicked the mucosa, was acquired on the surface of QCM- and SPR-sensing modules via flowing mucin aqueous solutions over the gold chips. Then, the changes of mass, viscoelasticity, and refractive index were evaluated during the interactions of TMD NS dispersions with the mucin layer.

#### Mass and Viscoelasticity Variation Studied by QCM

The principle of QCM is based on a piezoelectric effect (Reviakine et al., 2011). The variations of the resonant frequency (*F*) in odd overtones are measured to directly reflect the mass change of the materials on the surface of its sensing module via Sauerbrey equation (Mushi et al., 2014).

(1) $$\begin{array}{r} \Delta m=-C\Delta F \end{array}$$

Where *m* is the mass, *C* is the sensitivity constant of the quartz crystal (17.7 ng cm^2^/Hz for this study). The attenuation of oscillation amplitudes at a certain overtone is described as the dissipation (*D*), which gives information on the surface viscoelasticity (Yousefi and Tufenkji, 2016).

**Figure 2:**
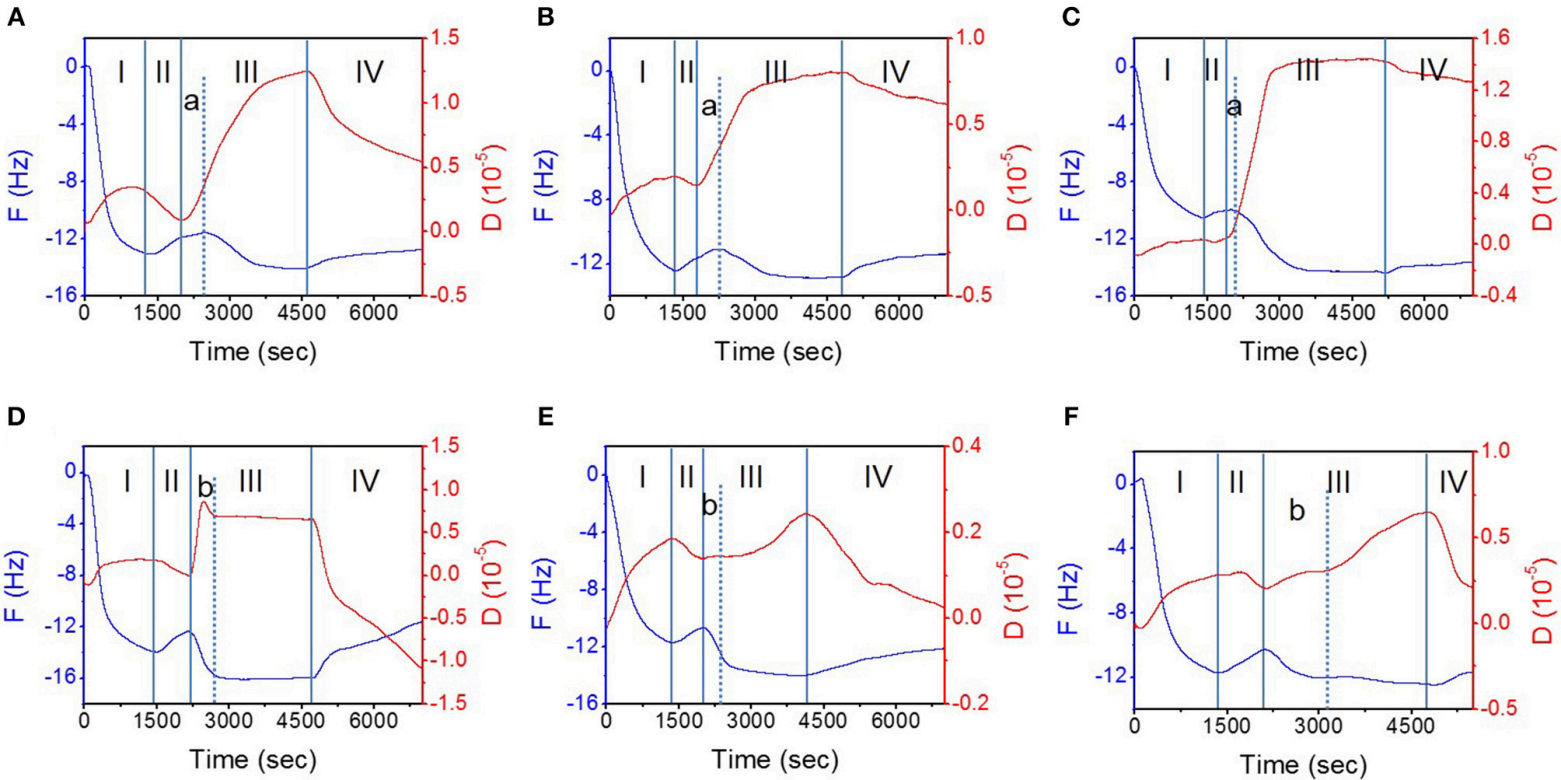
The interaction of mucin hydrogel and TMD NSs studied by QCM. **(A–F)** Depict typical variation of the *F* and *D* values with time during the interaction of mucin and SL MoS_2_, FL MoS_2_, SFL MoS_2_, SL WS_2_, FL WS_2_, and SFL WS_2_, respectively. The stages (I–IV) are introduced in “2.3. QCM study” of the “Materials and Methods” section.

Figure 2 shows the variations of *F* and *D* values over time when the interaction between the different TMD NSs and the mucin layer occurred on the surface of the gold film chip. In the different experimental stages (described in section QCM Study), the trend of *F* and *D* variation is the same. In stage I, when the mucin aqueous solution flowed over the gold chip surface, the *F* value descended while the *D* value ascended owing to the increase of the mass and viscoelasticity of the materials on the surface, which indicated the adsorption of mucin on the surface of the QCM sensing module and the formation of a mucin layer. In stage II, ultrapure water was introduced to remove the unstably combined mucin. The *F* value ascended as the *D* value descended because the slight desorption of mucin from the gold surface. The average weight of the mucin layer formed on the sensing module was 197.7 ng/cm^2^. In stage III, the *F* value decreased and the *D* value increased when TMD NS dispersions were flowed over the mucin layer, indicating an increased mass and viscoelasticity. In stage IV, an increase of the *F* value and a decrease of the *D* value were observed because of the desorption of the mucin and TMD NSs, which demonstrated a decrease of the mass and viscoelasticity. The main difference existed in stage III. When the MoS_2_ NS dispersions flowed over the mucin layer (Figures 2A–C), slight increases of the *F* values were recorded at the beginning (shown as “stage a”), and the *D* values continued to increase. During “stage a,” the adsorption and desorption of the MoS_2_ NSs, as well as the desorption of the mucin, occurred simultaneously, but the desorption rate of the MoS_2_ NSs and mucin was higher than the adsorption rate of the MoS_2_ NSs. The duration of the “stage a” change trend was SL MoS_2_ > FL MoS_2_ > SFL MoS_2_, indicating that the MoS_2_ NSs with higher flakiness had a smaller initial adsorption rate, and they were more prone to cause the desorption of the mucin. On the other hand, a “stage b” was divided at the beginning inside the stage III. When FL WS_2_ and SFL WS_2_ interacted with mucin (Figures 2E,F), the *D* value rose slowly in “stage b,” and then the rate of rising increased. At the same time, the *F* value declined in “stage b,” and then the rate of decline decreased. These results revealed that in “stage b,” FL WS_2_ and SFL WS_2_ experienced fast adsorption to the mucin but did not severely enhance its viscoelasticity. Inversely, in “stage b,” the *F* value declined quickly, while the *D* value of SL WS_2_ exhibited a very sharp increase followed by a slight decline, indicating that SL WS_2_ could quickly adsorb on mucin and severely affect its viscoelasticity.

**Figure 3:**
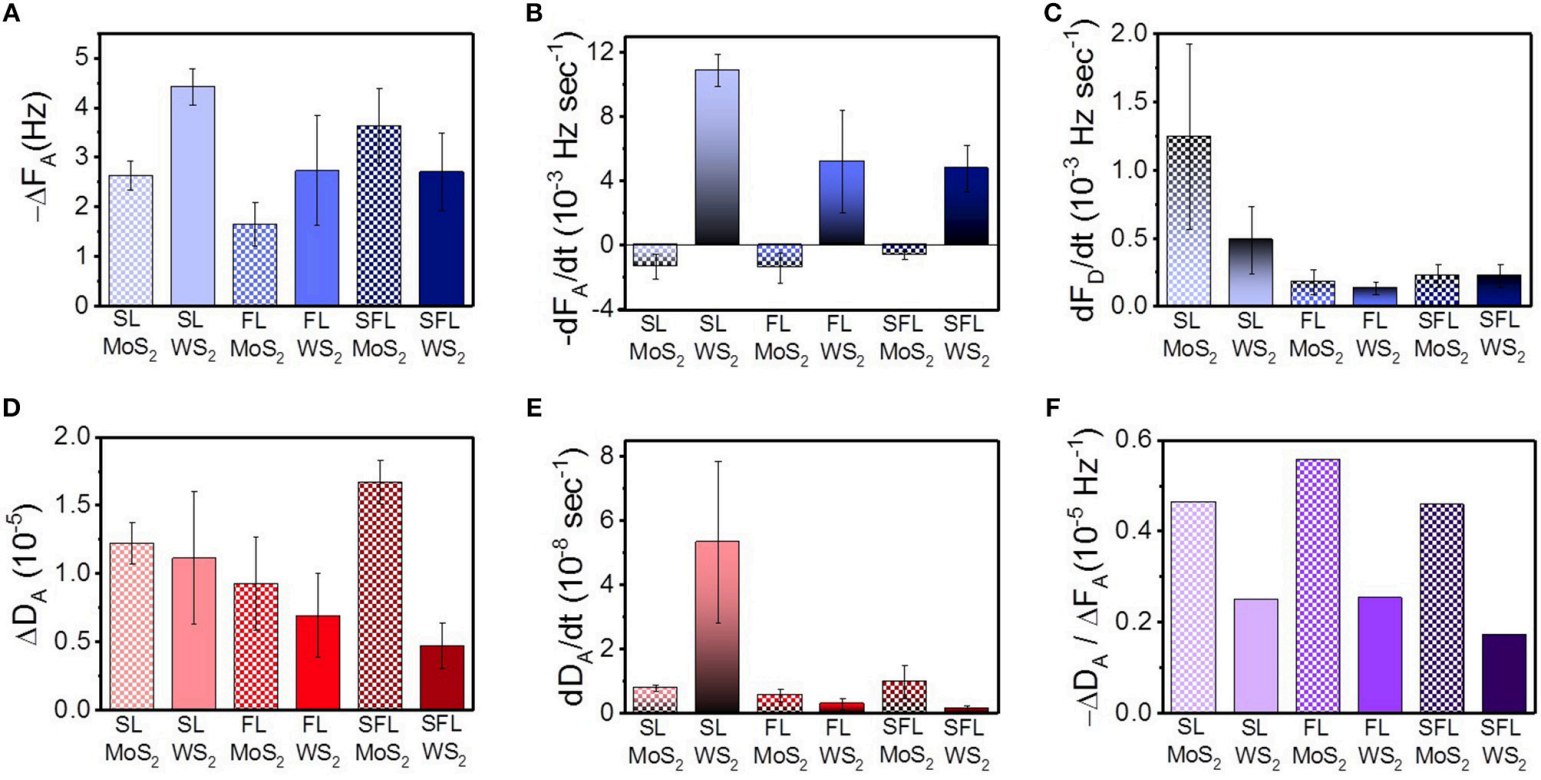
Important indexes of the interaction between mucin and the different TMD NSs studied through QCM. **(A)** total change in frequency in the adsorption; **(B)** initial rate of frequency change in the adsorption; **(C)** initial rate of frequency change in the desorption; **(D)** total change of dissipation value in the adsorption; **(E)** initial rate of dissipation change in the adsorption; **(F)** changes in the dissipation value associated with the change of frequency.

Figure 3 was plotted in order to clearly compare the indexes of the interaction between the TMD NSs and mucin. In Figure 3A, −Δ*F*_A_ is the total change in frequency in the adsorption stage, which was the negative value of the minimum *F* value minus the *F* value at its beginning. It can reflect the maximum variation of mass during the whole adsorption stage. The mass variation of SL WS_2_ was the highest among all of the TMD NSs, while the FL MoS_2_ was the lowest. −*dF*_*A*_/*dt* in Figure 3B is the initial rate of frequency change in the adsorption stage, which was defined as a variation of the *F* value in the first 150 s of the adsorption stage, revealing the rate of mass change at the initial stage of adsorption. The −*d*_*A*_/*dt* values of the WS_2_ NSs were positive, among which the value of SL WS_2_ was the highest, while the −*dF*_*A*_/*dt* values of the MoS_2_ NSs were negative. Similarly, in Figure 3C, −*dF*_*D*_/*dt* is the initial rate of frequency change in the desorption stage, defined as the variation of *F* value in the first 150 s of the desorption stage, reflecting the rate of mass change at the initial stage of desorption. SL MoS_2_ had the highest −*dF*_*D*_/*dt* value, while FL WS_2_ had the lowest. In short, the results indicated that SL WS_2_ exhibited the highest initial absorption rate and the maximum absorption amount, while the MoS_2_ NSs had a negative initial adsorption rate, suggesting the desorption of mucin with MoS_2_ at the beginning (stage a, as mentioned before). Additionally, the SL MoS_2_ exhibited the highest initial desorption rate.

The viscoelasticity changes of the protein hydrogel layer can be caused by the changes in the structure of protein (Wang et al., 2010). In Figure 3D, we defined Δ*D*_A_ as the total change in dissipation in the adsorption stage, i.e., the maximum *D* in the adsorption stage minus the *D* value at its beginning. This index reveals the influence of the TMD NSs on the structure of the mucin in the whole adsorption stage. SFL MoS_2_ was highest in this index, while SFL WS_2_ was the lowest. In Figure 3E, *dD*_*A*_/*dt* is the initial rate of dissipation change in the adsorption stage, which was defined as a variation of *D* value in the first 150 s of the adsorption stage. This index reflects the rate of viscoelasticity change and further reveals the intensity of the influence of the TMD NSs on the mucin structure at the initial stage of adsorption. The *dD*_*A*_/*dt* value of SL WS_2_ was much higher than that of the other TMD NSs, indicating that it could rapidly affect the structure of mucin. In Figure 3F, −Δ*D*_A_/Δ*F*_A_ is the variation of the dissipation value accompanying the change in unit frequency in the adsorption stage, which gives information about the viscoelasticity change caused by per unit mass change. The −Δ*D*_A_/Δ*F*_A_ value of FL MoS_2_ had the highest of all the TMD NSs, while the SFL WS_2_ had the lowest −Δ*D*_A_/Δ*F*_A_ value. In general, the −Δ*D*_A_/Δ*F*_A_ value of the WS_2_ NSs was smaller than that of the MoS_2_ NSs, indicating that when the TMD NSs in the same mass adsorbed onto the mucin, the WS_2_ NSs caused less influence on its viscoelasticity than the MoS_2_ NSs. Based on the above experimental results, suitable TMD NSs can be selected for specific biomedical applications as demanded.

#### Refractive Index Studied by SPR

**Figure 4:**
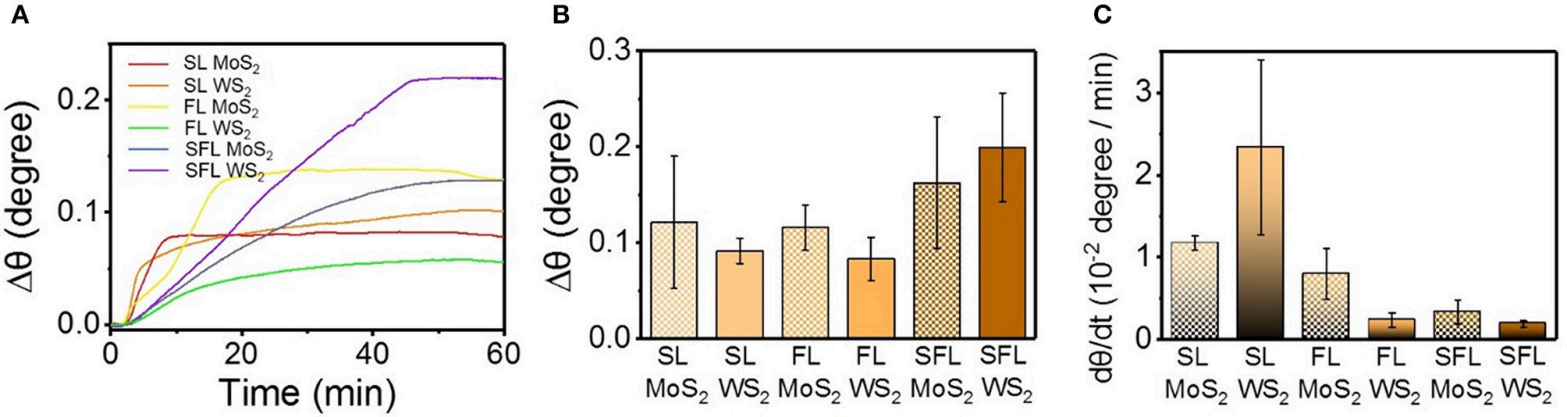
The interaction between mucin and the different TMD NSs studied by SPR method. **(A)** SPR angle change with time; **(B)** The change value of SPR angle; **(C)** Initial change rate of SPR angle.

The refractive index of materials on the surface of the sensing chip can be detected in real-time via SPR study (Homola, 2008; Liu et al., 2016), and the variation of the refractive index of the protein layer is closely related with the conformational changes of the protein (Dell'Orco and Koch, 2016), Figure 4A shows the variation of the SPR angle (θ) with time, and Δθ is the difference between the SPR angle at a certain time and the initial SPR angle. It can be found that SL MoS_2_, and FL MoS_2_ tended to achieve a quick saturation within 20 min, while the other four kinds of TMD NSs tended to achiever a slower saturation over 40 min. Δθ in Figure 4B is the total variation of θ during the adsorption as the maximum of θ minus the initial θ. *dθ*/*dt* that was introduced to exhibit the initial variation of θ (Figure 4C), which is defined as the variation of θ in the first 150 s of the adsorption stage. The *dθ*/*dt* value of SL WS_2_ was much higher than the other TMD NSs, and this result was consistent with the *dF*_A_/*dt* value of the adsorption in the QCM study. The *dθ*/*dt* value of the NSs with the same chemical constituents exhibited a decreasing trend with the lowered flakiness. The TMD NSs with higher flakiness tended to have higher Δθ and lower *dθ*/*dt*.

### The Interactions in the Aqueous Phase Studied by Spectroscopic Methods

#### UV Absorption Spectra

**Figure 5:**
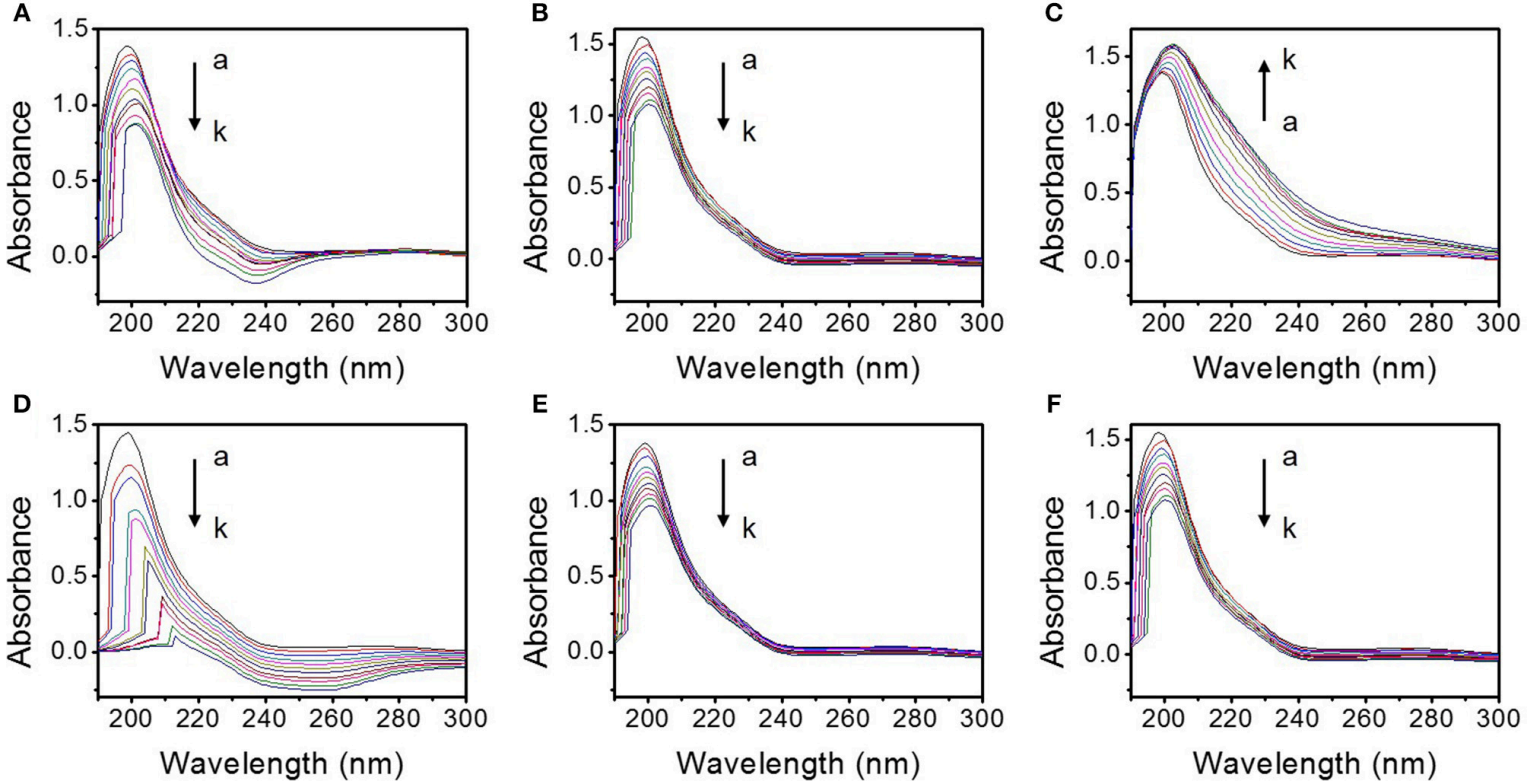
UV absorption spectra of the interaction system between different TMD NSs and mucin in the solution. **(A–F)** are the ultraviolet absorption spectra of the interaction system of mucin and SL MoS_2_, FL MoS_2_, SFL MoS_2_, SL WS_2_, FL WS_2_, and SFL WS_2_, respectively. a-k depict that the final concentrations of the TMD NSs are 0, 0.005, 0.01, 0.015, 0.02, 0.025, 0.03, 0.035, 0.04, 0.045, and 0.05 mg/mL, respectively.

The influences of the different TMD NSs on the UV absorption spectra of mucin are shown in Figure 5. With the addition of the TMD NSs, the variation of the absorption peak of peptide bonds (~200 nm) can be clearly observed. FL MoS_2_, FL WS_2_, and SFL WS_2_ caused smaller change of the UV absorption spectra than the other three TMD NSs. An obvious change at 240 nm was observed in the SL MoS_2_ and mucin interaction system. SL WS_2_ severely weakened the absorption peak of peptide bonds and made it significantly redshift, while the absorption peak of peptide bonds was redshifted and enhanced by the SFL MoS_2_. These experimental results indicated that the conformation of mucin was not significantly affected by FL MoS_2_, FL WS_2_ and SFL WS_2_, and the microenvironment of peptide bonds of mucin were severely changed in the presence of SL MoS_2_, SL WS_2_, and SFL MoS_2_. The experimental results are consistent with the *dD*_A_/*dt* value in the “QCM study” section.

#### Fluorescence Quenching Spectra

The chromophore residues of proteins, including tryptophan, tyrosine and phenylalanine, can serve as intrinsic fluorescent probes, and these aromatic amino acid residues are sensitive to their microenvironment. Therefore, fluorescence quenching behaviors of mucin by the TMD NSs were characterized. As shown in Supplementary Figure 3, with the increase of the TMD NS concentration, the fluorescence of mucin was significantly quenched. The SL MoS_2_, SL WS_2_, and SFL MoS_2_ exhibited higher quenching efficiency among the TMD NSs. This may result in stronger variation in the microenvironment of the chromophore residue of mucin, and possibly changes in its tertiary structure. The results are consistent with the change in UV-vis absorption spectra.

Shifts in the emission maximum wavelength of mucin with variations in the concentrations of the TMD NSs can be found in the normalized fluorescence spectra (Supplementary Figure 4). SL WS_2_ caused the largest blueshift, followed by SFL MoS_2_ and SL MoS_2_. This kind of shift may demonstrate some changes in the microenvironment of the aromatic amino acid residues, as well as variations of the protein tertiary structure. The blueshifts indicate a decrease in the hydrophilicity of the emission-active residues of mucin (Zamolo et al., 2018).

Fluorescence quenching mechanisms include static quenching and dynamic quenching. Dynamic quenching refers to the transfer of energy or electrons during the collision between the quenching agent and fluorescence active materials in the excited state, which does not affect the structure and activity of the protein. Static quenching refers to the quenching agent and fluorescence material interactions and generate a certain kind of ground-state complex without the emission of photons, and the fluorescence intensity is consequently reduced (Chi et al., 2010; Wang et al., 2011; Hao et al., 2017). Plots of fluorescence emission intensity *I*_0_/*I* vs. the TMD NSs were given as Supplementary Figure 5, where *I*_0_ and *I* represent the maximum fluorescence emission intensity of mucin in the absence and presence of the TMD NSs, respectively. When the final concentration of the TMD NSs was lower than 0.025 mg/mL, the fluorescence emission intensity exhibited a linear trend, which implies that the protein underwent a dynamic collisional quenching. With the higher concentration of the TMD NSs, the emission intensity of SL MoS_2_, SL WS_2_, FL MoS_2_, and SFL MoS_2_ exhibited an exponential trend, while that of FL WS_2_ and SFL WS_2_ maintained linearly. This reveals that when interacting with FL WS_2_ and SFL WS_2_, mucin kept dynamic quenching, but additional static quenching occurred when mucin interacted with SL MoS_2_, FL MoS_2_, SL WS_2_, and SFL MoS_2_. These results offer more evidence that SL MoS_2_, FL MoS_2_, SL WS_2_, and SFL MoS_2_ exerted a stronger influence on the conformation of mucin than FL WS_2_ and SFL WS_2_ did.

At low TMD NS concentrations, the fluorescence quenching was driven by diffusive transport, and the relative kinetic efficiency of the fluorescence quenching of mucin can be derived based on the non-equilibrium Stern-Volmer model (Kenry et al., 2016; Wani et al., 2018), as described in equation (1),

(2) $$\begin{array}{r} \frac{I_{0}}{I}=1+K_{SV}C_{Q} \end{array}$$

Where *K*_*SV*_ is the Stern-Volmer constant signifying the fluorescence quenching efficiency, and *C*_*Q*_ is the concentration of the quenching agent. The *K*_*SV*_ values are compared in Supplementary Figure 6. SL MoS_2_ and SL WS_2_ exhibited higher quenching efficiency than other TMD NSs. These two TMD NSs were the highest in flakiness, implying a higher relative surface area and resulting in a more efficient fluorescence quenching.

The relative strength and cooperativity of the interaction of the TMD NSs and mucin were further studied based on the quantification of several key parameters, including the binding dissociation constant *K*_*D*_, binding association constant *K*_*A*_ (the reciprocal of *K*_*D*_), and the Hill coefficient *n*. These parameters can be obtained through the Hill equations as follows:

(3) $$\begin{array}{r} Q=\frac{\left(I_{0}-I\right)}{I_{0}} \end{array}$$

(4) $$\begin{array}{r} \frac{Q}{Q_{\max}}=\frac{{C_{Q}}^{n}}{{K_{D}}^{n}+{C_{Q}}^{n}} \end{array}$$

**Figure 6:**
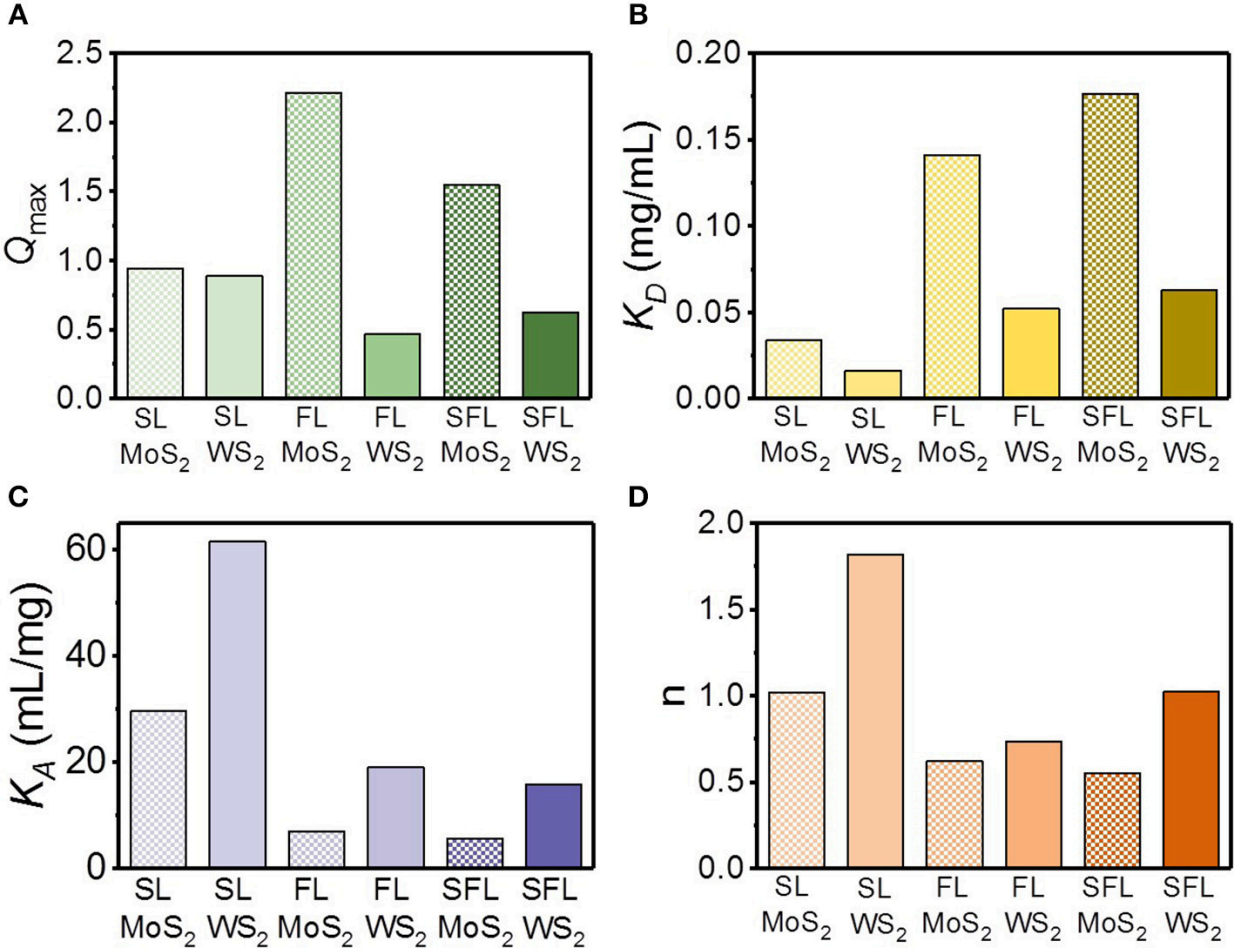
The estimation of relative strength and cooperativity of the interaction between mucin and different TMD NSs. **(A)** *Q*_max_; **(B)** dissociation constant *K*_*D*_; **(C)** association constant *K*_*A*_; **(D)** Hill coefficient.

Where *Q*_max_ is the saturation value of *Q*, which can be derived through non-linear fit of fluorescence quenching (Figure 6A; Supplementary Figure 7). *K*_*D*_ is the equilibrium binding dissociation constant, which describes the relative strength of the TMD NS–mucin interaction, and *n* is the Hill coefficient, which defines the cooperativity of the TMD NS–mucin association. As displayed in Figures 6B,C, the *K*_*D*_ value of the MoS_2_ NSs was higher than that of the WS_2_ NSs in the same hydrodynamic diameter. In addition, for the TMD NSs with the same element, the higher the flakiness was, the higher *K*_*A*_ value was. These results imply that the WS_2_ NSs tend to be easier to associate with mucin than the MoS_2_ NSs and that the NSs with higher relative surface area (the SL NSs) have a stronger association with mucin. The Hill coefficient *n* describes the binding cooperativity between the TMD NSs and mucin (Figure 6D). The SL WS_2_-mucin complex exhibited cooperative binding with positive cooperativity (*n* > 1), implying that an increased amount of mucin adsorbed onto the surface of SL WS_2_ will enhance the binding strength between the proteins and the surface. In contrast, the FL MoS_2_-mucin, FL WS_2_-mucin, and SFL MoS_2_-mucin complexes displayed anti-cooperative binding with negative cooperativity (*n* < 1). This indicates that the more mucin that is absorbed on the surface of these TMD NSs, the weaker binding strength will be.

#### CD Spectroscopy

**Figure 7:**
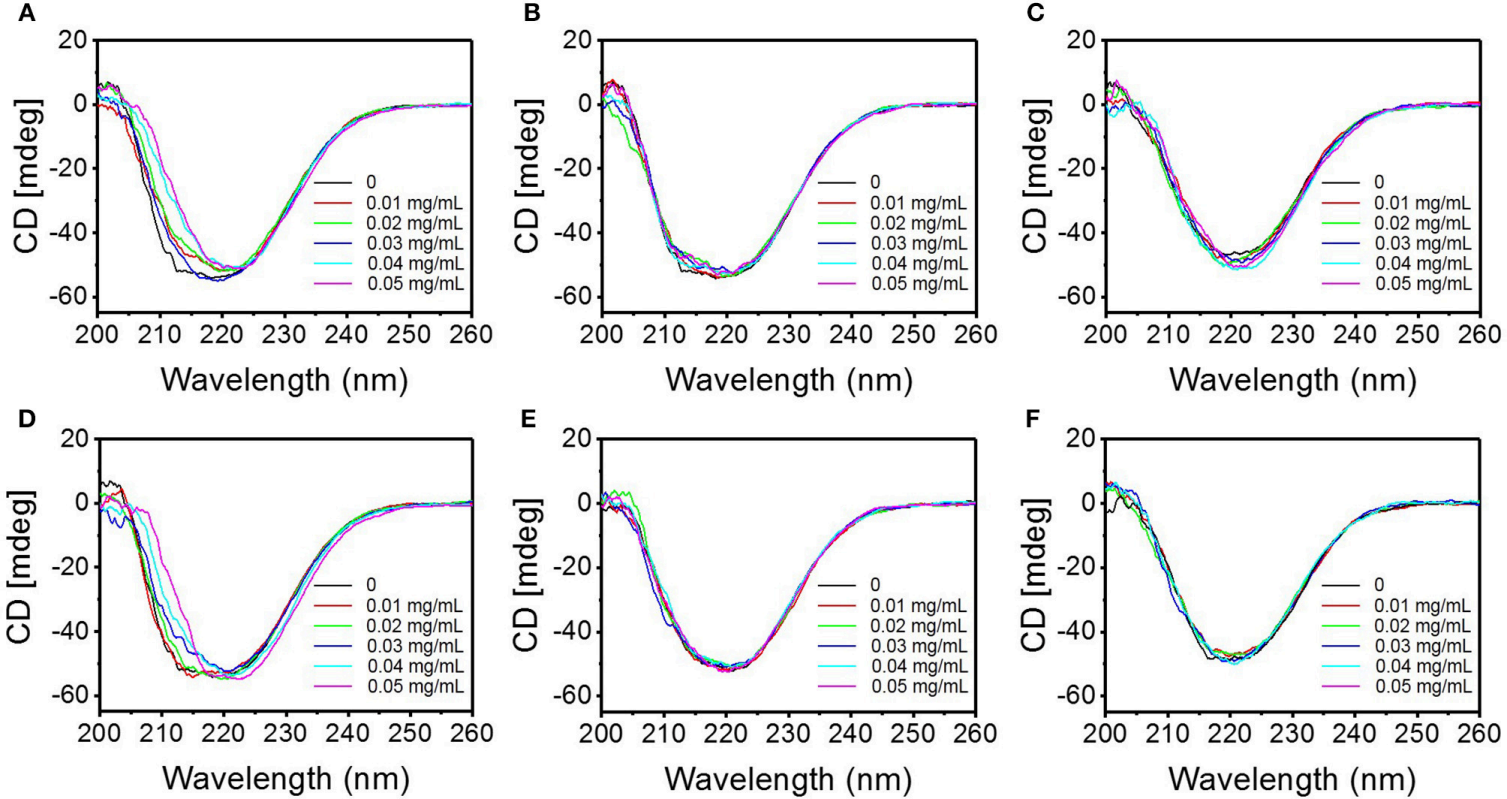
Circular dichroism spectra of the interaction system between the different TMD NSs and mucin in solution. **(A–F)** are circular dichroism spectra of the interaction system of mucin and SL MoS_2_, FL MoS_2_, SFL MoS_2_, SL WS_2_, FL WS_2_, and SFL WS_2_, respectively.

CD spectroscopy is an effective tool for rapid determination of secondary structure of proteins (Greenfield, 2006). It is widely used to determine the fold of purified proteins and the influence on their conformation or stability caused by the microenvironment changes (Murtaza et al., 2018). Far ultraviolet CD spectra (200–260 nm), which reveal the variations in the secondary structure of the proteins resulted from the interaction between proteins and other substances (Kenry et al., 2017), were investigated to describe the interaction between the TMD NSs and mucin (Figure 7). With the increasing of the concentration of TMD NSs, the CD spectra of the interaction systems between mucin and FL MoS_2_, FL WS_2_, and SFL WS_2_ did not exhibit much variation, while SL MoS_2_ and SL WS_2_ caused the CD spectra of mucin to redshift, and SFL MoS_2_ slightly enlarged the peak of CD spectra. These experiment results were in accordance with the trends of UV spectra variation as mentioned.

The analysis of secondary structure variation of mucin was conducted via CD Pro software. As shown in Supplementary Figure 8, sharp variation of the percentage of secondary structures in each typical sort did not occur when the final concentration of TMD NSs increased from 0.01 to 0.05 mg/mL. With the increasing of the concentration of TMD NSs, SFL MoS_2_ increased the percentage of α-helix, and decreased the percentage of β-sheet. In contrast, the interaction of mucin with other TMD NSs made its percentage of α-helix higher, and β-sheet lower, and SL MoS_2_ and SL WS_2_ caused larger-scale variation. These results gave the information of conformational change of mucin during its interactions with the TMD NSs.

### Mechanism of the Interactions Between the TMD NSs and Mucin

**Scheme 1:**
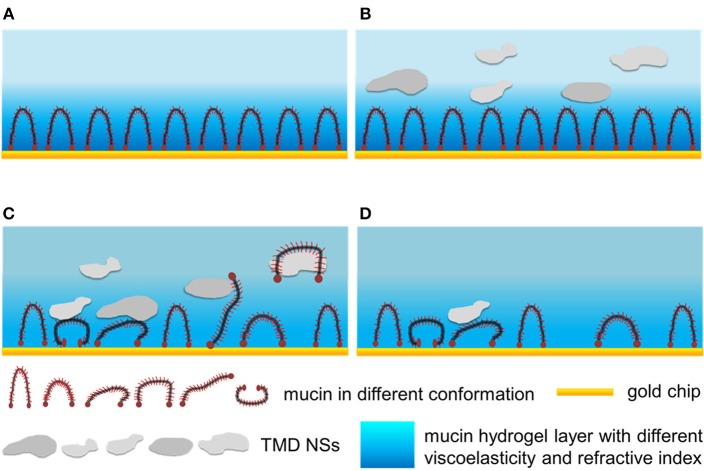
The interactions between TMD NSs and mucin in the mimic mucosa on the surface of QCM-D and SPR sensor chips. **(A)** The formation of mucin hydrogel layer. **(B)** TMD NSs adsorbed on the mucin layer. **(C)** TMD NSs interacted with the mucin layer, resulting in the conformational changes of mucin, and the formation of mucin-TMD NS complexes. **(D)** TMD NS-mucin complex fell off from the gold surfaces after thorough washing.

According to the results mentioned above, here a possible mechanism of the interactions between the TMD NSs and mucin was proposed, as shown in Scheme 1. When the mucin solution flowed over the gold chip, the protein adhered onto the gold surface and formed a hydrogel layer with water (Scheme 1A). When the dispersions of TMD NSs flowed over the mucin hydrogel layer, the mass, viscoelasticity and refractive index of the materials on the gold chip surface variated. These results indicated that the TMD NSs adsorbed and interacted with the mucins, resulting in their conformational changes, and the variation of their amino acid residues' microenvironments (Schemes 1B,C). In this stage, SL WS_2_ can quickly adsorb on the mucin layer, and the viscoelasticity variations of the mucin layer caused by the MoS_2_ nanosheets were more intense than those caused by the WS_2_ nanosheets. It was observed in the spectroscopic probing that SL MoS_2_, SL WS_2_, and SFL MoS_2_ caused higher the conformational changes of mucin than the other three TMD NSs. TMD NSs interact with mucin molecules by hydrophobic effect. The TMD NS-mucin complex probably fell off from the gold surfaces after the thorough washing, thus resulting in the increased *F* value and the decreased *D* value (Scheme 1D). In this stage, SL MoS_2_ exhibited the highest initial desorption rate.

Mucin consists of a protein core and glycosyls in its common structure. The protein core is arranged into distinct regions. The central glycosylated region is comprised of a large number of tandem repeats that are rich in serine, threonine and proline, while the regions with an amino acid composition more representative of globular proteins are located at the terminals. The central glycosylated region is highly glycosylated consisting of ~80% carbohydrates primarily *N*-acetylgalactosamine, *N*-acetylglucosamine, fucose, galactose, and sialic acid, and the glycosyls are arranged in a “bottle brush” configuration around the protein core (Bansil and Turner, 2006). The central glycosylated region is weakly negatively charged owing to the carboxyl groups. As the TMD NSs are also negatively charged, the adsorption of TMD NSs can be hindered by the electrostatic repulsion. On the other hand, the TMD NSs with a large surface energy were able to be adsorbed on the mucin layer via the non-covalent interaction, such as the van der Waals' force, hydrogen binding, and hydrophobic interaction, and some of the TMD NSs interacted with the mucin proteins, to form a NS-mucin complex. The NS–mucin complex and the large-sized TMD NSs desorbed from the sensor surfaces following the washing buffer step, thus resulting in an increased *F* value and a decreased *D* value (Figure 2).

## Conclusions

In summary, we demonstrated the interactions between mucin and TMD NSs using QCM-D, SPR, and spectroscopic methods, including UV absorption spectroscopy, fluorescence quenching spectroscopy, and CD spectroscopy. The results indicated that the TMD NSs adsorbed on the mucin layer and affected its viscoelasticity and refractive index. SL WS_2_ exhibited the highest initial absorption rate and the maximum absorption amount, while SL MoS_2_ exhibited the highest initial desorption rate. During the adsorption, the viscoelasticity variations of the mucin layer caused by the WS_2_ nanosheets was weaker than that caused by the MoS_2_ nanosheets. Furthermore, the conformational changes of mucin caused by SL MoS_2_, SL WS_2_, and SFL MoS_2_ were higher than those from other TMD NSs. This work provides insights on the interactions between TMD NSs and mucin. Suitable TMD NSs can be selected for specific biomedical applications based on the findings in this study.

## Author Contributions

BL, RH, and RS designed research. BL and TY performed research. All the authors analyzed data and wrote the paper and read and approved the final manuscript.

### Conflict of Interest Statement

The authors declare that the research was conducted in the absence of any commercial or financial relationships that could be construed as a potential conflict of interest.
