# Supplementary material for: Interactions of Transition Metal Dichalcogenide Nanosheets With Mucin: Quartz Crystal Microbalance With Dissipation, Surface Plasmon Resonance, and Spectroscopic Probing
Source: Front Chem. 2019 Mar 29;7:166. doi: 10.3389/fchem.2019.00166 (PMC6449427; doi:10.3389/fchem.2019.00166)
Supplement: Supplementary file 1 [file Data_Sheet_1.docx]

Supplementary Material

Interactions of Transition Metal Dichalcogenide Nanosheets with Mucin: QCM-D, SPR and Spectroscopic Probing

Boshi Liu^2,3^, Tao Yu^2^, Renliang Huang^1*^, Rongxin Su^2,4*^, Wei Qi^2,4^, Zhimin He^2^

^1^Tianjin Key Laboratory of Indoor Air Environmental Quality Control, School of Environmental Science and Engineering, Tianjin University, Tianjin 300072, P. R. China

^2^State Key Laboratory of Chemical Engineering, Tianjin Key Laboratory of Membrane Science and Desalination Technology, School of Chemical Engineering and Technology, Tianjin University, Tianjin 300072, P. R. China

^3^Tianjin Key Laboratory of Modern Chinese Medicine, College of Pharmaceutical Engineering of Traditional Chinese Medicine, Tianjin University of Traditional Chinese Medicine, Tianjin 300193, PR China.

^4^Collaborative Innovation Center of Chemical Science and Engineering (Tianjin), Tianjin 300072, P. R. China

***Correspondence:**Renliang Huang
tjuhrl@tju.edu.cn
Rongxin Su
surx@tju.edu.cn

.

# Supplementary Figures


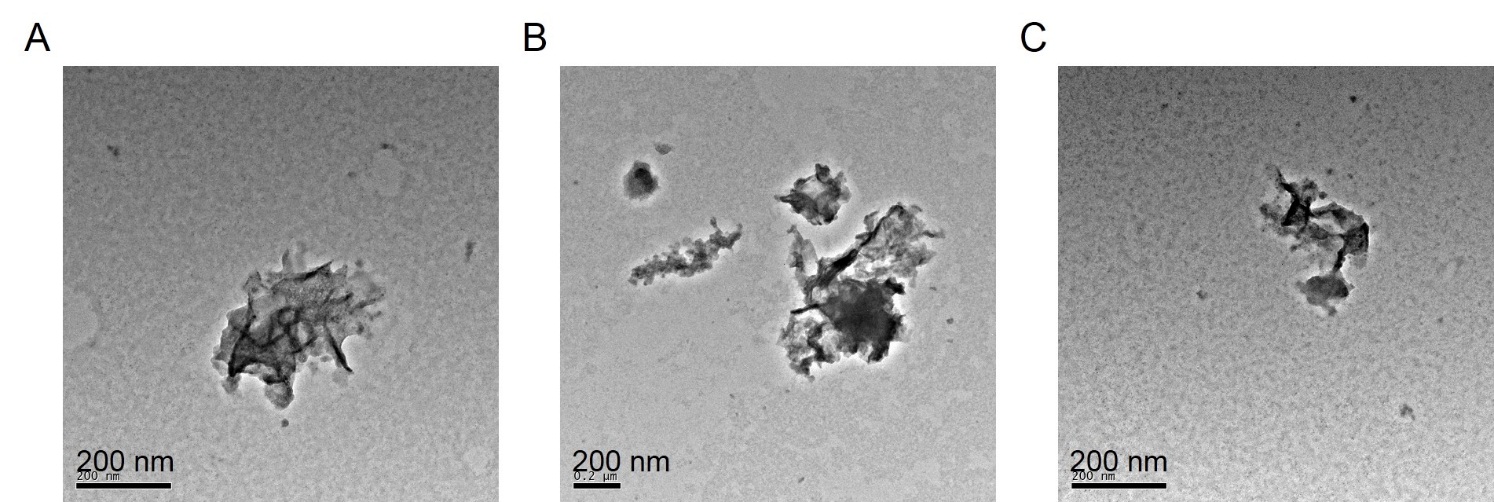


**Supplementary Figure S1.** TEM pictures of MoS_2_ nanosheets. (A: SL MoS_2_; B: FL MoS_2_; C: SFL MoS_2_)


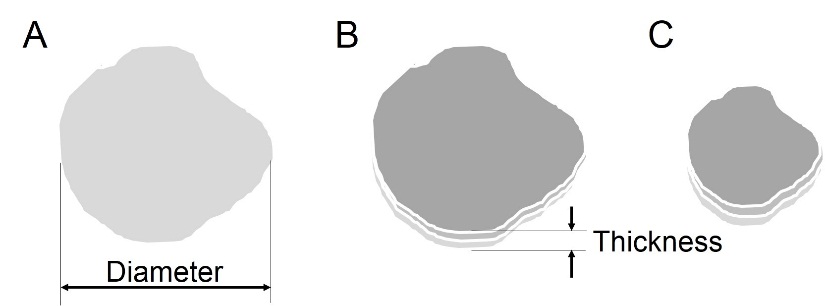


**Supplementary Figure S2.** The morphology sketch of the transition metal dichalcogenide nanosheets (A: single-layered TMD NSs; B: few-layered TMD NSs; C: small few-layered TMD NSs).


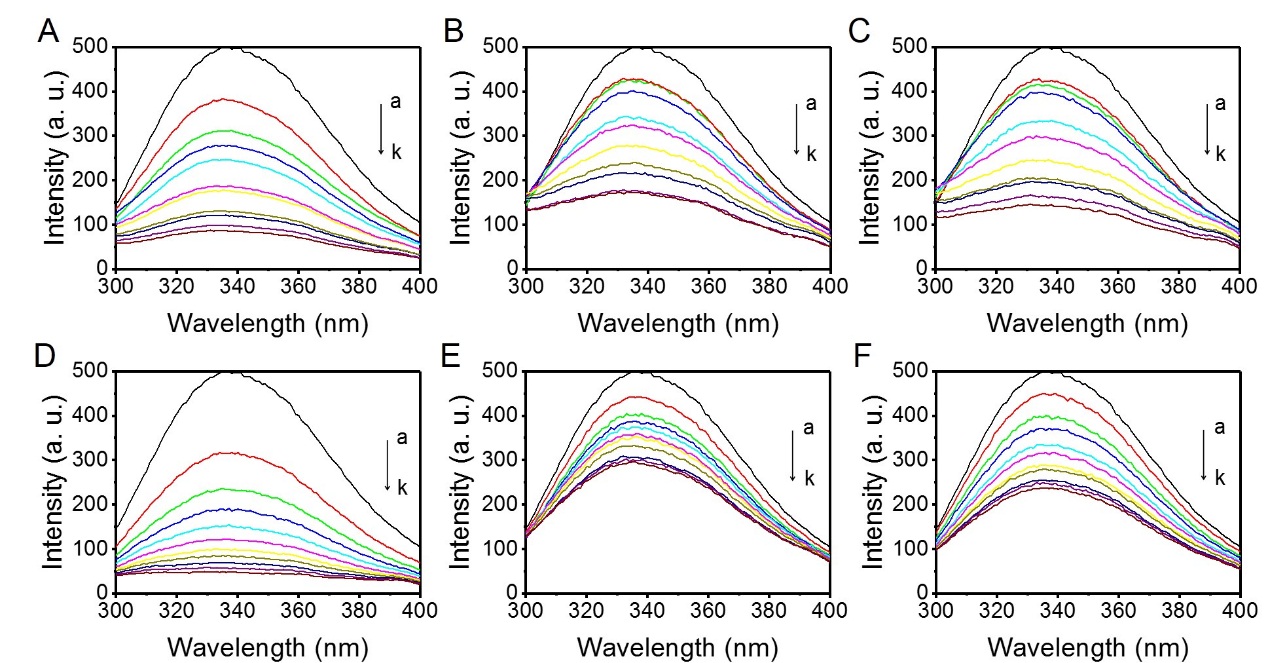


**Supplementary Figure S3.** Fluorescence quenching spectra of the interaction systems between TMD NSs and mucin in aqueous phase. A−F are fluorescence quenching spectra of the interaction system of mucin and SL MoS_2_, FL MoS_2_, SFL MoS_2_, SL WS_2_, FL WS_2_, SFL WS_2_, respectively. a−k depicts the final concentrations of TMD NSs were 0, 0.005, 0.01, 0.015, 0.02, 0.025, 0.03, 0.035, 0.04, 0.045, 0.05 mg/mL, respectively.


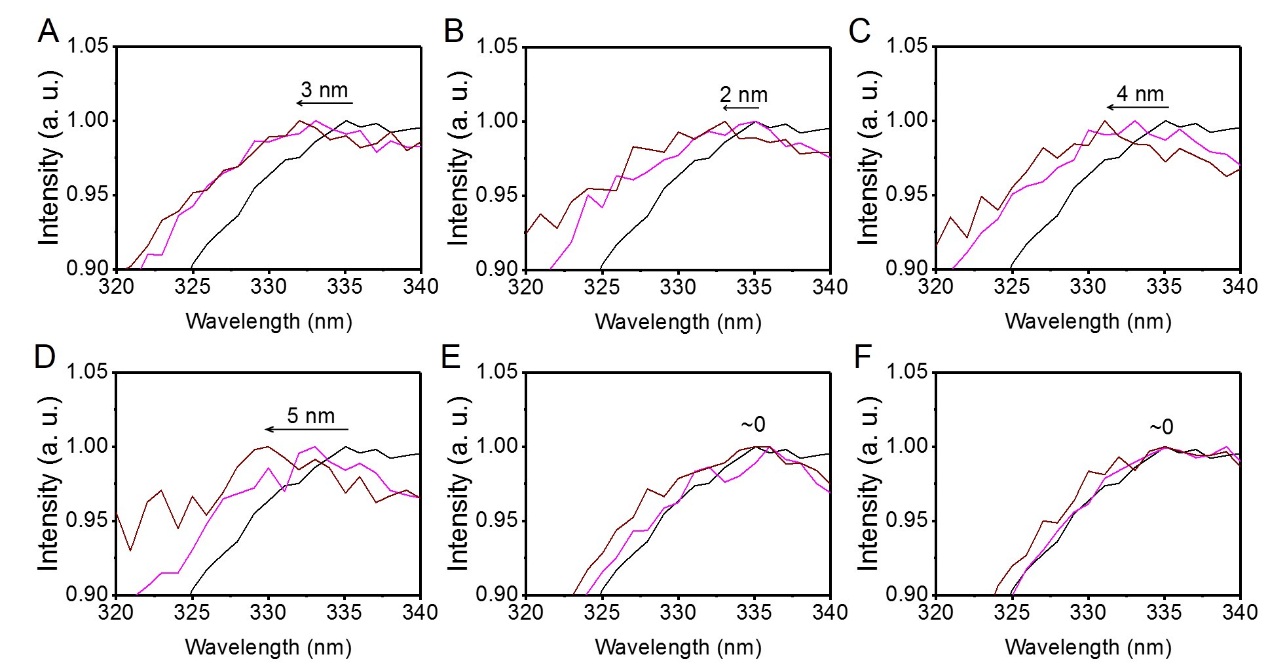


**Supplementary Figure S4.** Normalized fluorescence quenching spectra of the interaction systems between TMD NSs and mucin in aqueous phase. A−F are the normalized fluorescence quenching spectra of the interaction system of mucin and SL MoS_2_, FL MoS_2_, SFL MoS_2_, SL WS_2_, FL WS_2_, SFL WS_2_, respectively. Black, purple and brown lines are normalized fluorescence quenching spectra with TMD NSs’ final concentrations as 0, 0.025, and 0.05 mg/mL, respectively.


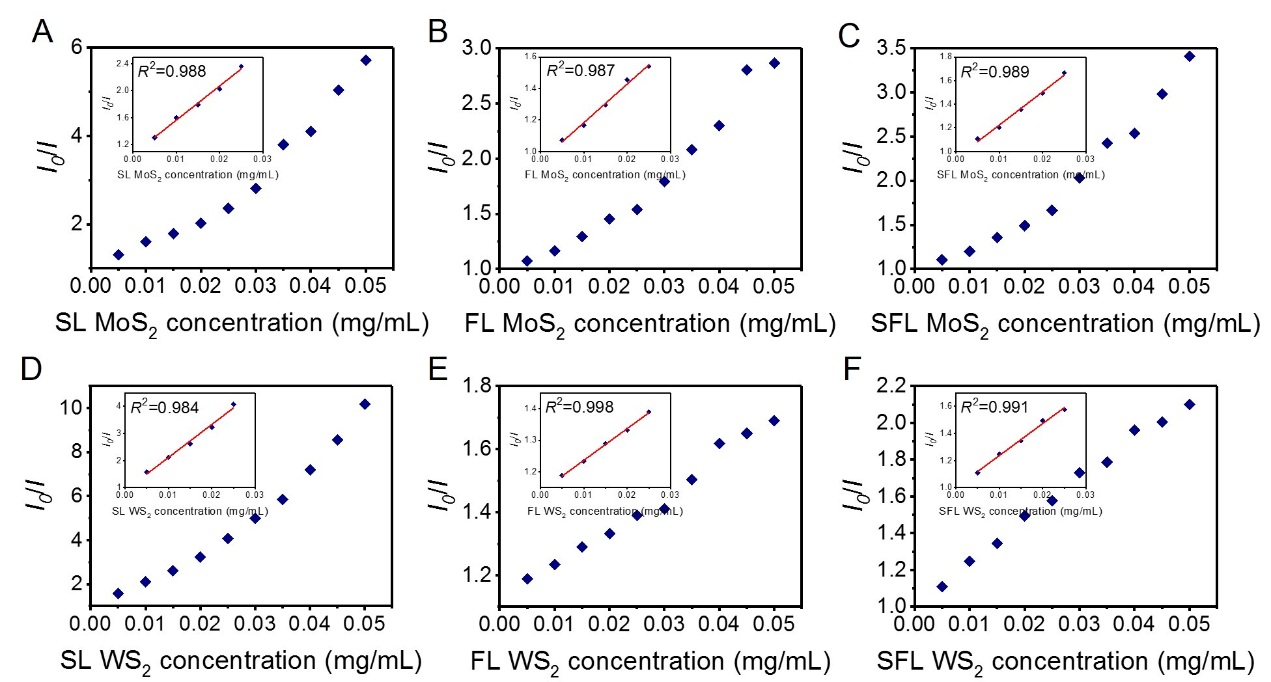


**Supplementary Figure S5.** The study of fluorescence quenching mechanism. A−F are Stern-Volmer plots of the interaction system of mucin and SL MoS_2_, FL MoS_2_, SFL MoS_2_, SL WS_2_, FL WS_2_, SFL WS_2_, respectively.


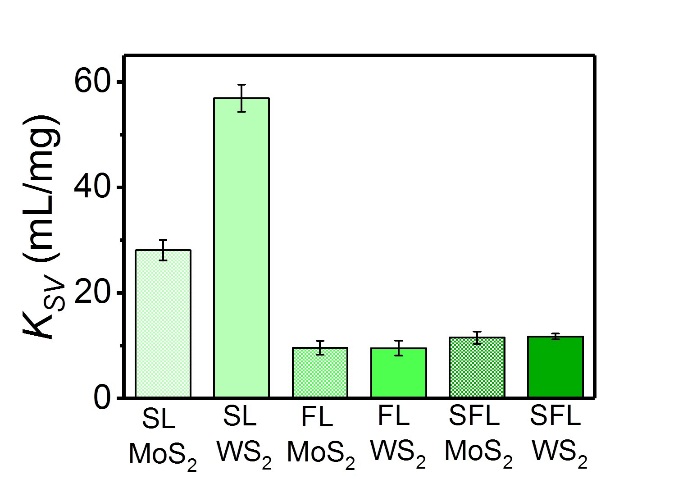


**Supplementary Figure S6.** *K_SV_* values of the TMD NSs to mucin.


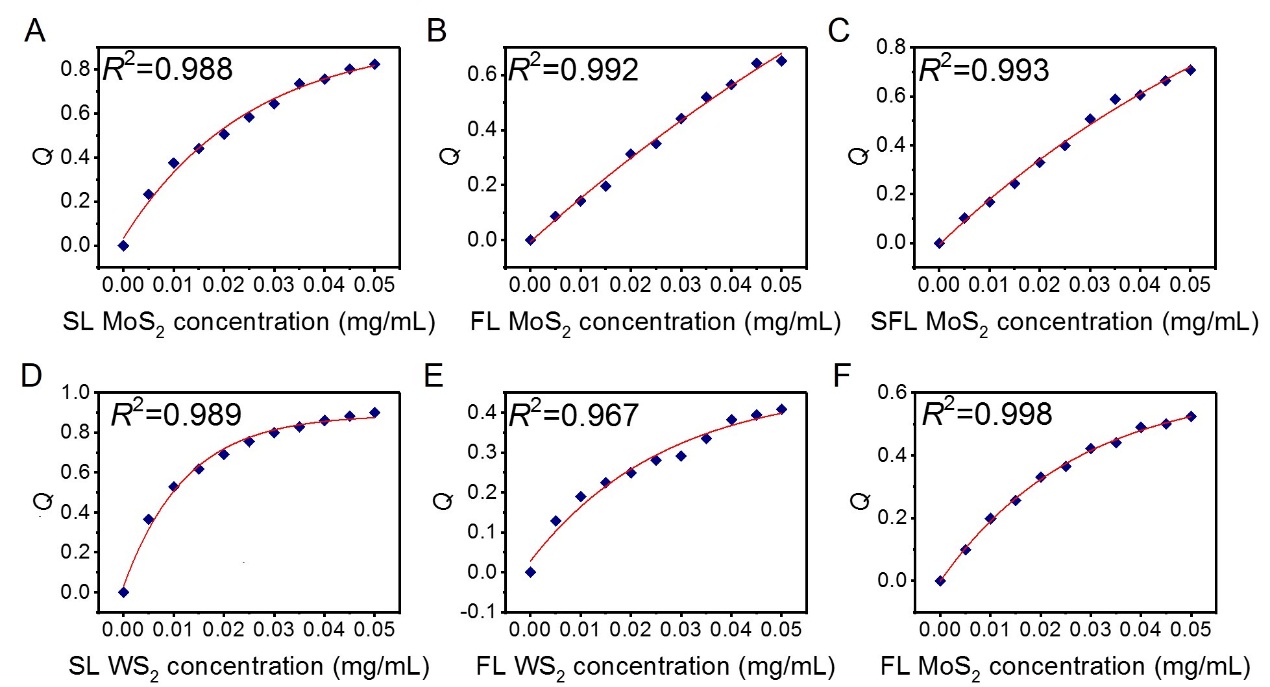


**Supplementary Figure S7.** Hill plots of the interaction system between different transition metal dichalcogenide nanosheets and mucin in solution. A−F are the plots of *Q* value vs the concentration of SL MoS_2_, FL MoS_2_, SFL MoS_2_, SL WS_2_, FL WS_2_, SFL WS_2_ in their interaction system with mucin, respectively.


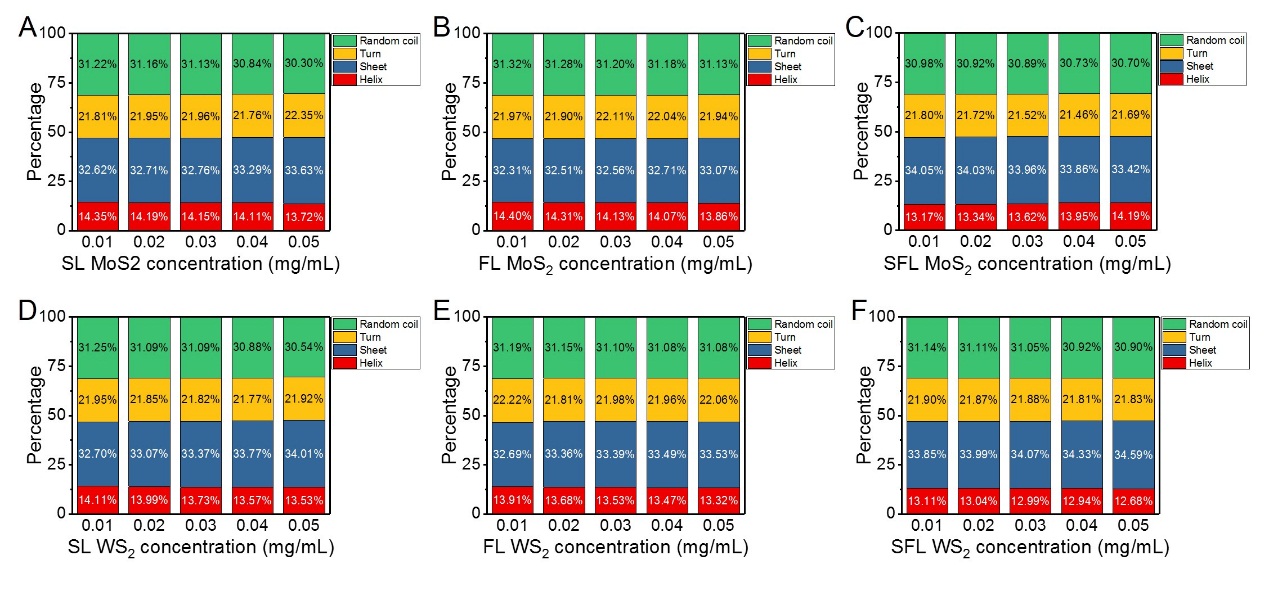


**Supplementary Figure S8.** The analysis of secondary structure change through CD Pro software. A−F depict the secondary structure change of mucin in its interaction system with SL MoS_2_, FL MoS_2_, SFL MoS_2_, SL WS_2_, FL WS_2_, SFL WS_2_, respectively.
